# Supplementary material for: Transcriptomic Analysis Provides Insights into the Differential Effects of Aluminum on Peanut (Arachis hypogaea L.)
Source: Genes (Basel). 2022 Oct 10;13(10):1830. doi: 10.3390/genes13101830 (PMC9602356; doi:10.3390/genes13101830)
Supplement: Supplementary file 1 [file genes-13-01830-s001.zip › genes-1904878-supplementary.pdf]

Supporting Information

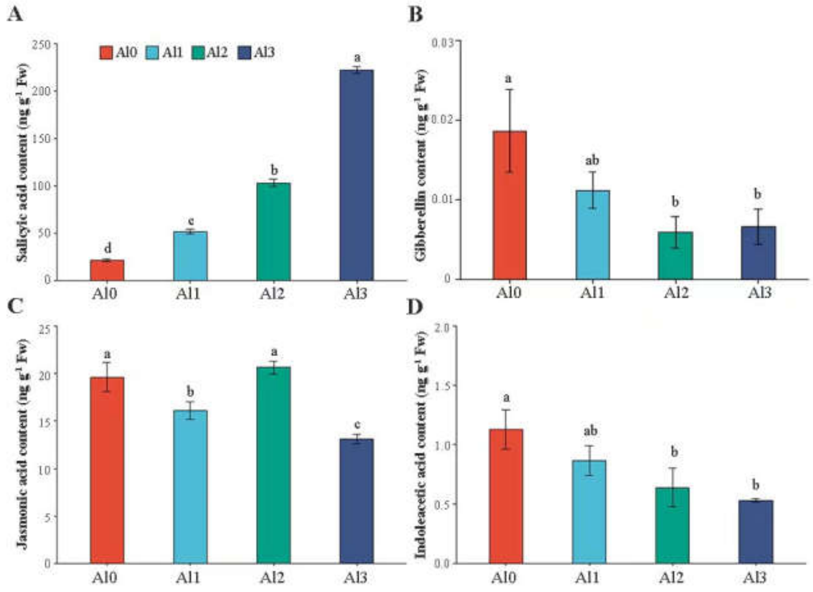

Figure S1. Effect of different Al^{3+} concentrations on the plant hormone content. The effect of Al treatments on salicylic content (**A**). The effect of Al treatments on gibberellin content (**B**). The effect of Al treatments on jamonic acid content (**C**). The effect of Al treatments on indoleacetic acid content (**D**). Al0, Al1, Al2 and Al3 represent 0 (pH 6.85), 1.25 (pH 4.03), 2.5 (pH 3.85) and 5 (pH 3.69) mmol/L $AlCl_3.18H_2O$ solutions, respectively. Data are means (±SE), n=3. Marking the same letters means $P \geq 0.05$ (LSD), there is no significant difference, the difference between different letters means $P<0.05$ (LSD), and the difference is significant.
